# Gender based violence (GBV) coordination in a complex, multi-crisis context: a qualitative case study of Lebanon’s compounded crises (2019–2023)

**DOI:** 10.1186/s13031-023-00543-8

**Published:** 2023-10-23

**Authors:** Philomena Raftery, Jinan Usta, Ligia Kiss, Jennifer Palmer, Mazeda Hossain

**Affiliations:** 1 Department of Global Health & Development, London School of Hygiene and Tropical Medicine, Keppel Street, London, UK; 2 Department of Family Medicine, American University Beirut, Beirut, Lebanon; 3 Centre for Women, Peace & Security, London School of Economics and Political Science, Houghton Street, London, UK

**Keywords:** Gender-based violence (GBV), GBV coordination, Compounded emergencies, Public health emergencies, COVID-19 pandemic, Beirut Port explosion, Localization

## Abstract

**Background:**

Since 2019 Lebanon has faced multiple compounded crises. Political and social instability, the COVID-19 pandemic, and the Beirut Port explosion, alongside the influx of refugees related to the ongoing Syrian conflict, have resulted in a nationwide economic emergency. In the context of the humanitarian response to the Syrian conflict, the UN and government-led gender-based violence (GBV) task force has coordinated the sub-sector since 2012. The compounded crisis, however, created new challenges for GBV coordination and service delivery, which we explore in this paper. We highlight lessons for strengthening GBV coordination in Lebanon and other complex emergencies.

**Methods:**

We conducted 29 remote in-depth interviews, reviewed key policy documents and observed seven GBV task force meetings. We analysed and presented our findings across three key themes: context-relevant and adaptable coordination mechanisms; coordination to support GBV service delivery; and stakeholders’ roles, legitimacy and power.

**Results:**

Parallel response frameworks developed to address the multiple crises, created a complex humanitarian architecture within an increasingly challenging operating context, with some perceived inefficiencies. Positively, coordination was integrated under the established government-UN interagency system and the GBV task force maintained GBV sub-sector coordination. The task force was commended for effectively adapting to the evolving context, including working remotely, maintaining essential GBV services, assessing the compounded crises’ impact on programming and adjusting accordingly, and harmonising guidance, tools and approaches. The importance of ensuring a government co-led response was highlighted by both UN and government informants, who pointed to examples where marginalising government leadership compromised coordination effectiveness and sustainability. The participation of local actors had become increasingly important but more difficult, with the impact of the various crises, and remote modalities, challenging service delivery and staff wellbeing.

**Conclusion:**

Experiences from Lebanon highlight the essential role of government leadership in coordination; the value of investing in local GBV capacity; the significance of effective national, subnational and intersectoral coordination to support service delivery and address cross-cutting GBV issues; the importance of targeted interventions to support marginalised populations; and the need to prioritize the well-being of front-line staff during crisis response. In Lebanon, and other complex crises, donors are encouraged to increase flexible, multiyear funding for GBV coordination and services, while women-led organizations should be at the forefront of recovery efforts, contributing to a more equitable society.

**Supplementary Information:**

The online version contains supplementary material available at 10.1186/s13031-023-00543-8.

## Introduction

Over the past four years Lebanon has faced multiple, compounded crises (2019–2023). The progressive collapse of the economy brought on by political and social instability, the Coronavirus-19 (COVID-19) pandemic, and the Beirut Port explosion, alongside the longer-term influx of refugees related to the ongoing conflict in Syria, has generated a nationwide emergency. Such complex humanitarian emergencies can perpetuate gender-based violence (GBV), which is recognised as a global health, human rights, and protection issue [1–5]. Effective GBV coordination ensures that GBV response services (including health, psychosocial, legal, and socio-economic support) are available to survivors and that prevention and mitigation measures are implemented to reduce incidents of GBV [6]. In Lebanon, the GBV coordination system, originally established in 2012 to respond to the influx of Syrian refugees, had evolved and expanded to become the main coordination mechanism in the country for GBV actors and had markedly enhanced systems and services for addressing GBV throughout the protracted crisis [7–10]. Previous research applying ‘the framework for effective GBV coordination’ noted that GBV coordinators had successfully forged relationships across diverse stakeholders to create an effective coordination structure within the complex Lebanese context [7, 11]. In parallel, during the past decade, Lebanon has made substantial progress in reforming the legal framework to protect women from GBV, with civil society playing a vital role in advocating for change [8]. However, many gaps remain and challenges towards achieving gender equality include weak female participation in parliament and the sectarian legal system that does not grant equal rights to women [12]. Since 2019, the compounded crises have created new challenges for GBV coordination and service delivery, which the task force had to overcome. In this paper, we explore GBV coordination during this period, and highlight lessons for strengthening GBV coordination in Lebanon and other complex emergencies.

The Syrian crisis that began in 2011 has had a profound economic, environmental and social impact on Lebanon [13–15]. By 2023, around 1.5 million Syrian refugees remain displaced in Lebanon, constituting almost a quarter of the total population [16, 17]. As Lebanon has not signed the 1951 Refugee Convention, legal protections for refugees are limited and over the course of the protracted crisis, refugee policies became increasingly hostile, making it difficult for Syrians to access services, maintain legal residency, and find employment [14, 15, 18, 19]. As official refugee camps were not established for Syrian refugees, they are dispersed across informal tented settlements, and among the urban population [8, 15, 17, 18]. Despite recent efforts to ease legal residency processes, the registration rate remains low at 31% as of 2021, with only 16% of Syrians over 15 years of age holding legal residency [17, 20].

Corruption and financial mismanagement has left Lebanon the third most indebted country globally [21, 22]. Lebanon’s economic collapse is often attributed to the refugee crisis, but analysts argue that the country’s sectarian political system and the government’s failure to implement reforms were driving factors predating the Syrian conflict [21, 23–25]. Protests demanding reforms and an end to corruption started in October 2019, coinciding with a default on foreign debt and a 90% currency devaluation [26–28]. The COVID-19 pandemic hit Lebanon in February 2020, and intermittent lockdowns and mitigation measures worsened the situation, challenging the already weak public health system [26, 29]. Further compounding this dire set of circumstances, a massive explosion at Beirut’s port on 4 August 2020 devasted areas of the city, caused more than 215 deaths and over 6,000 injuries, and displaced more than 300,000 people, including 81,000 women of reproductive age and 48,000 adolescents [30, 31]. Estimated costs to Beirut’s infrastructure were around USD 3.1 billion, while the impact on the economy was estimated at approximately USD 920 million [32].

Since 2019, Lebanon has descended into a severe economic and financial crisis, ranked among the top 10 most severe global crises by the World Bank [33]. A once middle income country, by 2022 the majority of the population were deprived of adequate access to basic services that were mostly privatised, including healthcare, education, clean water and electricity [26, 34]. In 2022, 82% of the Lebanese population (3 million people) were estimated to be living in multidimensional poverty and 90% of Syrian refugee families were living in extreme poverty [17, 35]. Hyperinflation, which was estimated to average 145% in 2021, increased food prices by 400% [27, 34]. Throughout 2021, fuel and electricity shortages caused nationwide blackouts, disproportionately affecting marginalized groups [36]. In parallel, the government gradually ended its fuel subsidy program, causing the cost of fuel to drastically increase [36]. Limited supplies of subsidized drugs and medical equipment strained hospitals operating under COVID-19 conditions, resulting in some closures and ward shutdowns [21, 26]. This was further exacerbated by a failure to pay healthcare workers causing many staff, including doctors and nurses, to emigrate [37]. Furthermore, in October 2022, the country declared the first cholera outbreak since 1993 [38]. Rent costs have increased, leading to increased eviction threats and evictions that have particularly affected refugees and women-led households in urban areas [17, 20, 34].

Caretaker administrations have led Lebanon for most of the last three years and have delayed governance reforms, causing public frustration and a mass exodus of Lebanese professionals [27, 28]. Meanwhile, international donors, distrustful of the Lebanese government, have withheld assistance. Hezbollah has opposed transparent investigation into the cause of the Beirut Blast, exacerbating divisions as the public demands accountability and justice [28, 39]. In addition, host-refugee tensions and security incidents have increased, and the Lebanese Armed Forces are overstretched and underpaid, causing fears of a breakdown in peace [14, 28]. National elections in May 2022 saw Hezbollah’s seats in parliament reduced, altering the balance of power in parliament with some hope for political reform [40, 41]. Since then, however, Lebanon has faced a political vacuum with no president and only a caretaker government, while the country’s economic crisis continues to worsen [40, 41].

The compounded crises have disproportionately affected women and GBV rates have intensified [42]. Many low-income families have been pushed into extreme poverty, and prolonged periods of isolation during COVID-19 lockdowns, along with job losses exacerbating stress and negative coping strategies, have increased GBV risks [19–21, 29, 34]. In 2020, calls to emergency hotlines increased substantially in both number and severity as compared with the previous year [43]. Female migrant workers, subjected to a Lebanese sponsorship system, were abandoned by employers, leaving them reliant on basic humanitarian support and highly vulnerable to GBV and exploitation [34, 44, 45]. There are also increasing reports of transactional sex, particularly affecting low-income women, with significant mental health consequences [44, 46]. An increase in child marriage has been reported among Syrian refugees in Lebanon, exacerbated by the prolonged school closures due to COVID-19 in 2020-21 [49–49]. The move to technology-based communication created new risks, including online harassment and cyberstalking and some reports indicate that GBV against lesbian, gay, bisexual, transgender, intersex, queer, and other (LGBTIQ+) individuals has increased [50]. According to the 2022 GBVIMS annual report for Lebanon, 95% of GBV survivors were female, with children accounting for 17% of reported incidents [51]. The main nationalities involved in GBV incidents were displaced Syrians (74%), followed by Lebanese nationals (23%) and other nationalities (3%). Physical assault (33%) and psychological/emotional abuse (32%) were the most reported types of GBV, followed by sexual violence (16%), with most incidents perpetrated by an intimate partner (54%) [51]. Psychosocial support, health services, livelihood services, and case management were the main accessed and referred services and the report indicated a need for increased outreach to persons with disabilities (PwD) and other marginalised groups to ensure their access to GBV services [51].

By 2022, Lebanon had multiple humanitarian response frameworks in operation. The Ministry of Social Affairs (MOSA) alongside the United Nations (UN) and partners coordinated the response to the Syrian refugee crisis through the multi-year Lebanese Crisis Response Plan (LCRP) [13, 20]. The LCRP, covering 2022–2023, remained the primary response framework in the country, receiving over 87% of humanitarian funding [52]. Throughout 2020-22, the Ministry of Public Health (MOPH) and the World Health Organization (WHO) coordinated the public health response to the COVID-19 pandemic [53]. In the absence of a government response to the Beirut Blast, the United Nations Office for the Coordination of Humanitarian Affairs (OCHA) took the lead, launching a Flash Appeal to respond to immediate needs [54]. To complement these initiatives, in August 2021, a 12-month multi-sectoral Emergency Response Plan (ERP) was developed (and updated in 2022) to assist vulnerable Lebanese, Palestinians, and migrant workers not covered by the LCRP, including continued response to the COVID-19 pandemic [34]. Also in 2021, the ‘Reform, Recovery and Reconstruction Framework’ (3RF) costing USD 2.5 billion was implemented by the World Bank, UN, and EU to comprehensively respond to the Beirut Blast [55]. In parallel, a government-led, World Bank-supported project, the Emergency Crisis and COVID-19 Response Social Safety Net Project (ESSN), was established with a budget of USD 246 million to provide basic assistance to 786,000 vulnerable Lebanese people impacted by the socio-economic crisis and to support the development of a comprehensive national social safety net system [56, 57]. After years of negotiations, the International Monetary Fund (IMF) reached an agreement with Lebanon in April 2022 for a four-year funding bailout, with approval contingent on government reforms [28]. However, by March 2023, nearly a year after the IMF agreement was signed, Lebanese officials had not undertaken the necessary reforms to activate the 4-year financing recovery program, considered crucial for Lebanon to begin emerging from the economic and financial crisis [58].

Against this contextual backdrop, in this study we explored how the humanitarian coordination system and the GBV task force flexed to meet the expanding humanitarian needs, while adapting to overcome the particular challenges of the multiple, aforementioned crises. We examined how the crises affected coordination dynamics and perceived effectiveness, support for service delivery and the roles, legitimacy and power of different stakeholders. Building on previous research on humanitarian and GBV coordination including experiences from Lebanon’s protracted crisis [7, 11, 59], this study offers valuable lessons to enhance GBV coordination in Lebanon and other complex emergencies.

## Methods

### Study design and sampling

We conducted a case study over a 16-month period (2021–2022) to explore GBV coordination during Lebanon’s compounded crisis (2019–2023). We used mixed qualitative methods including a review of key policy documents, observation of seven GBV coordination meetings and 29 remote in-depth interviews with a range of GBV and humanitarian stakeholders. Both purposive sampling and snowball sampling were used to ensure a diverse range of relevant organizations and a balance of national and international stakeholders were represented in interviews [60]. Key informants comprised GBV experts from MOSA [1], UN agencies [4], INGOs [5], sub-national coordinators [4], national and local organizations [7] and academia [1] as well as UN coordinators of other related sectors/working groups (Interagency, Health, Clinical Management of Rape (CMR), Gender, Protection, Child Protection (CP) and Education) [8].

### Data collection and analysis

Semi-structured interviews were conducted using topic guides that were iteratively updated to include emerging themes (Additional File 1: Qualitative Interview Guides) and digitally recorded before transcription and analysis. Documents relating to GBV coordination and the humanitarian response included strategic plans, guidelines, reports and meeting minutes, among others (Additional File 2: Table 2: Documents included in the analysis). The lead researcher (PR) attended seven national-level remote GBV coordination meetings as a participant observer to gain a deeper understanding of coordination dynamics and the roles of stakeholders. A data verification workshop was held in May 2022 by the lead researcher (PR) in Beirut, which engaged members of the GBV task force, to validate interpretations and clarify any data gaps or uncertainties. Framework analysis [61] was used to analyse data, assisted by Nvivo 12 software [62]. Data from different sources were validated across multiple data sources, thereby increasing the validity and reliability of the results. Interviews were conducted until saturation was reached on several themes.

## Results

**Fig. 1:**
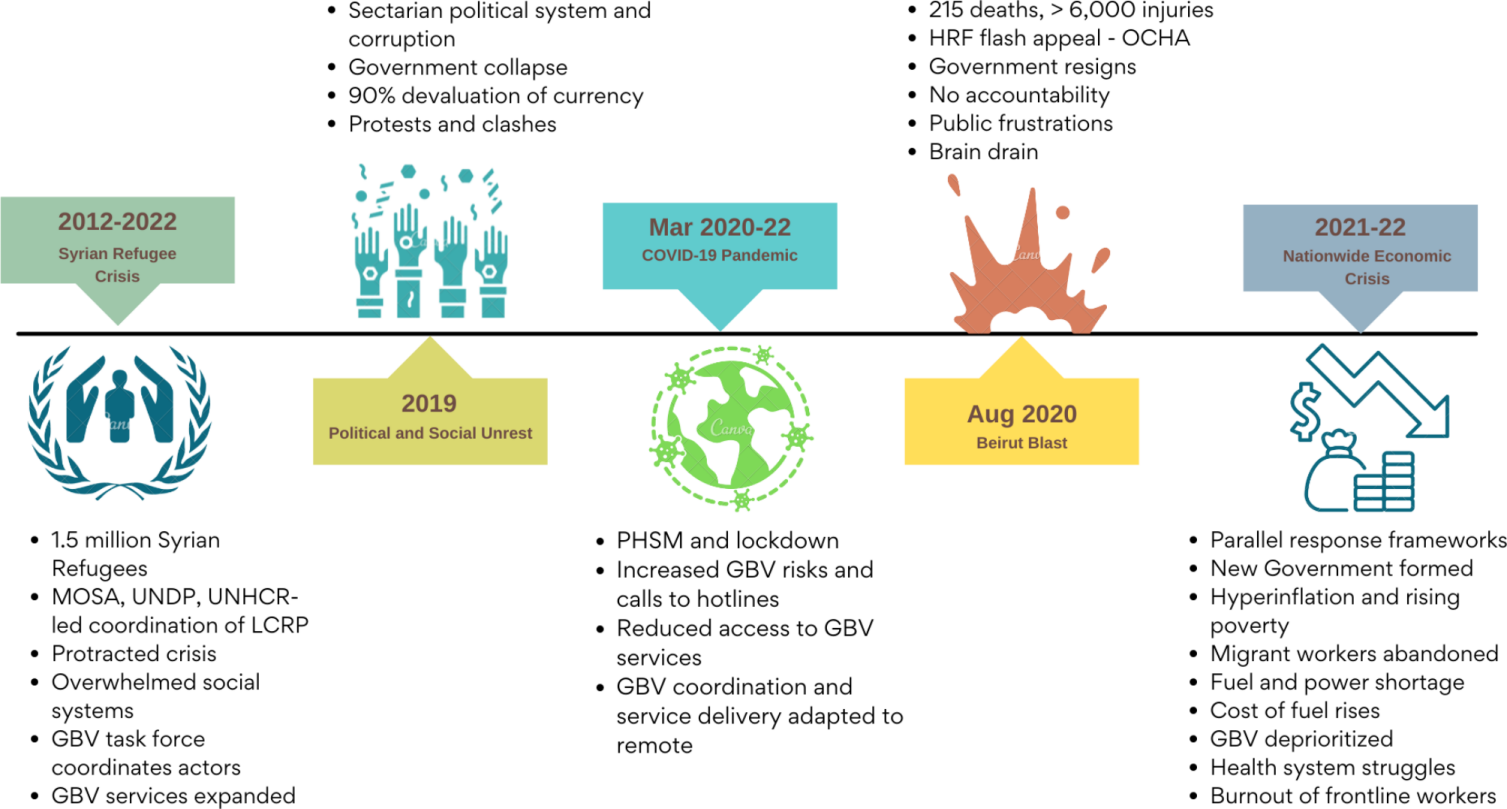
Lebanon’s compounded crises and key issues emerging within each crisis that aggravated the situation in the country and affected GBV coordination and service provision

We present our findings across three themes below: (1) context-relevant and adaptable GBV coordination mechanisms; (2) coordination to support GBV service delivery; and (3) stakeholders’ roles, legitimacy and power. Figure 1 presents the various crises in Lebanon and the issues associated with the specific events that aggravated the situation and affected GBV coordination and service provision. Table 1 outlines the challenges faced and solutions implemented by the GBV task force during the different phases of the compounded crises alongside the positive aspects of GBV coordination leveraged to address each challenge.

**Table 1:** Challenges faced and solutions implemented by the GBV task force during the different phases of the compounded crises and the aspects of GBV coordination leveraged to address each challenge

Emergency situation and Timeframe	Challenge	Solutions implemented by the GBV Task Force	Aspect of GBV coordination leveraged
Multiple crises 2019–2023	Parallel response frameworks developed to address the multiple crises, created a complex humanitarian architecture	• Coordination across all frameworks integrated at the sector level under the established LCRP interagency system • GBV task force maintained GBV sub-sector coordination • Implemented the Lebanon Aid Tracking initiative in 2023 for tracking GBV funding implementation across all organization’s - comprehensive of all humanitarian and development funding received in Lebanon including LCRP, ERP, and others	• National coordination
COVID-19 Pandemic and Public health and social measures 2020–2022	Transition of coordination meetings to virtual modality	• Continued monthly remote meetings at national and sub-national level • Strengthened the interface between the national and sub-national coordination through communication, coordinating and experience sharing	• National and sub-national coordination
		• Conducted a multi-stakeholder 2020 assessment on the impact of COVID-19 on GBV • Issued a report on the impact of COVID-19 on GBV survivors, focusing on minority groups such as LGBTIQ + communities • Published regular gender and COVID-19 policy briefs with recommendations for policy makers and practitioners to ensure a gender-equitable response	• Data and information management
	Reduced access to GBV services and transition of service delivery to remote modalities	• Developed guidelines for GBV response and risk mitigation during COVID-19 • Provided harmonized guidance and tools that supported actors at the field level to adapt to remote modalities and respond to the pandemic • Developed a training manual for health care providers to provide remote services to GBV survivors • Delivered trainings on provision of remote case management • Established an emergency hotline through which women and girls could reach security forces • Local actors supported women with safety plans and phone credit to call the hotline	• Intersectoral coordination • Support for service provision
	Reduced uptake of CMR services due to fear of contracting COVID-19 at public hospitals	• Provided alternative solutions including referral for CMR to INGOs rather than government run hospitals	• Support for service provision
	Negative COVID-19 test required by GBV survivors before being admitted to safe shelters (52)	• GBV and health partners allocated funding for testing GBV survivors • COVID-19 testing costs for GBV survivors incorporated in the ERP	• Intersectoral coordination
	Difficulties providing remote MHPSS services for GBV survivors	• Provided group psychosocial support via WhatsApp using a mixed approach of chats, voice messages and live calls • Prioritised high-risk cases for in-person services	• Support for service provision
	Increased GBV and child marriage in adolescent girls	• IRC initiated remote PSS programs for adolescent girls in Akkar and Bekaa, facilitating access via providing girls with phones and engaging outreach volunteers, with safety precautions taken to prevent additional risks including regular safety audits, and PSEA risks addressed through training of outreach volunteers	• Support for service provision
Beirut Blast Aug 2020	Major disaster on top of Syrian crisis, Economic crisis and COVID-19 pandemic	• Maintained MOSAs co-leadership of the national GBV task force	• Reinforce government leadership of coordination
		• Sub-national coordination mechanism leveraged in Beirut and Mount Lebanon • GBV actors accessed funding for GBV projects through HRF flash appeal	• Sub-national coordination • Prioritization and resources
Nationwide economic crisis including Fuel and power shortages 2021–2023	Fuel and power shortages	• Conducted an assessment in August 2021 to gauge the impact of Lebanon’s fuel and electricity crisis on GBV programming. Showed that 27% of providers reported severe impact on their capacity to deliver services, and 96% reported difficulties for service users to access GBV providers. Local providers implemented measures to ensure service continuity, with increased support for communication and transportation costs (38).	• Support for service provision • Localisation
	Increasing GBV risks and deepening vulnerability of marginalized groups alongside budget restrictions limiting initiatives to support LGBTIQ + survivors, PwDs, and those requiring MHPSS	• Targeted efforts to mobilize funding to support vulnerable populations to access to life-saving services including LGBTIQ + community, migrant workers, adolescent girls, PwDs • GBVIMS data used to assess the impact of the compounded crises and adapt GBV coordination and services • Used data to advocate for funding, develop policy briefs, inform programming and service coverage • Used the 2021 16 Days of Activism to raise awareness on increasing GBV risks, expanding vulnerable populations, and the importance of continuing to prioritize and fund GBV • Interagency working group developed a policy brief to highlight the needs of PwDs and identify policy and programming gaps. It provided recommendations for key stakeholders to ensure more inclusive and specialized services and interventions for PwD in the response (70). • Efforts to strengthen the use of the GBV Information Management System (GBVIMS) for Lebanese, Palestinian, migrant, and other nationalities, ensuring that outreach and support were inclusive of all nationalities	• Targeted support for marginalised groups • Support for service provision • Prioritization and resources • Intersectoral coordination • Data and information management
	Increased GBV cases among children and child marriage	• GBVIMS steering committee issued a report that made a series of recommendations on safeguarding and responding to the needs of adolescent girls and child survivors including improved coordination across the protection sector and strengthened programming focused on adolescent girls • Several agencies implemented approaches to engage with religious leaders on child marriage and GBV task force held a dedicated meeting to share approaches and good practice • GBV and CP held joint coordination meetings to discuss issues and approaches to address GBV among children including access to services and prevention activities • Developed a guidance note on Care and Support for Child Survivors of GBV • UNICEF and UNHCR developed decision tree on referrals of child survivors of GBV to case management services • In 2023, GBV organizations worked with MOSA to review and update the National Plan for the Prevention and Response of Child Marriage in Lebanon which was developed in 2020 but never officially launched	• Data and information management • Intersectoral coordination • National coordination • Support for service provision • Reinforce government leadership of coordination
	Lebanese currency devaluation and fluctuating exchange rates affected cost of delivering services	• Increased GBV sub-sector funding request for 2022 by 20% compared with previous years • Advocated for inclusion of GBV within ERP • Jointly with protection and CP, National GBV coordinators organised bilateral donor briefings with several donors (Spain, Norway, Italy, Switzerland, Canada, Netherlands, Denmark), to brief donors on sectoral trends and analysis and to highlight critical advocacy messages, especially the need for increased and multi-year funding.	• Prioritization and resources • Intersectoral coordination
	Deprioritization of GBV prevention and response across government and affected communities and GBV actors faced challenges in engaging women and girls with conventional GBV services	• UNFPA piloted implementation of cash assistance in case management and assessed the impact to guide further expansion • Local actors worked with survivors on medium to long term livelihoods plans • MOSA Protection coordinator co-leading GBV task force and advocated with government	• Prioritization and resources • Reinforce government leadership of coordination • Support for service provision
	Migrant workers abandoned by employers and left reliant on humanitarian assistance	• Included funding needs of migrant workers in ERP • Worked with Migrant Sector to support a mapping survey of migrant domestic workers’ main challenges and urgent needs	• Targeted support for marginalised groups
	Progress on institutionalising GBV services within government run social development centers (SDCs) threatened and lack of sustainability	• Capacity building and support to strengthen the role of national and local NGOs in service provision	• Support for service provision
	Provision of CMR services hampered by fuel and power shortages, as well as lack of payment for healthcare workers and low rates of GBV referrals from health service providers’	• Support to strengthen the role of national and local NGOs in service provision • Enhanced referrals and collaboration with health sector and CMR working group to engage front line workers	• Support for service provision • Intersectoral coordination
	High turnover of humanitarian actors and loss of skilled and educated Lebanese workers	• Reinforced government leadership of the humanitarian response • Maintained MOSAs co-leadership of the GBV task force • Included local and national organization’s as co-leads of sub-national coordination	• Reinforce government leadership of coordination • Localisation
	Staff burnout in local organization’s, threats and concerns about GBV staff safety and difficulties continuing service delivery	• Assessed the impact on frontline workers and highlighted findings with donors and other stakeholders • Local providers implemented adaptive measures to ensure the continuity of GBV services including providing support to GBV survivors for communication and transport • Local actors provided staff with psychosocial support	• Prioritization and resources • Support for service provision • Support for frontline staff
	Escalating levels of psychological and mental health issues, and a rise in substance abuse causing heightened risks of GBV	• MHPSS, Protection, GBV and CP working groups collaborated to implement a series of trainings for front line workers to enhance identification and safe referral of persons with MHPSS needs to relevant service providers. Sessions included self-care components.	• Intersectoral coordination • Support for service provision
	Lack of access to smartphones, phone credit, and internet connectivity challenged communication for GBV survivors	• Local actors allowed women and girls to charge phones in local offices • Service providers increased funding for communications and transport for women and girls • The UNHCR Innovation Service introduced a Digital Gender Inclusion and Innovation Bootcamp to equip refugee-led and community-based organizations with the necessary skills to bridge the digital gender gap in their communications	• Support for service provision • Localisation

Abbreviations: CMR: Clinical Management of Rape,COVID-19: Coronavirus-19, CP: Child Protection, CSO: Civil Society Organization’s, ERP: Emergency response plan, GBV: Gender Based Violence, GBVIMS: Gender Based Violence Information Management System, INGO: International non-governmental organization, LCRP: Lebanon Country Response Plan, LGBTIQ+: Lesbian, gay, bisexual, transgender, intersex, queer, +, MHPSS: Mental health and psychosocial support, MOSA: Ministry of Social Affairs, NGO: Non-governmental organization, PSEA: Protection from Sexual Exploitation and Abuse, PwD: Persons with Disabilities, SDC: Social Development Centre, UNDP: United Nations Development Programme, UNFPA: United Nations Population Fund, UNHCR: United Nations High Commissioner for Refugees, UNICEF: United Nations Children’s Fund

### Context-relevant and adaptable GBV coordination mechanisms

Since 2019, the Lebanese context had changed drastically, requiring the humanitarian and GBV coordination mechanisms to adapt to multiple, interconnected, crises within an increasingly challenging operating context. Parallel emergency response frameworks, developed to respond to the multiple crisis, were intended to be complementary but created a complex humanitarian architecture that actors had to navigate. Additionally, reporting had to be conducted against the different individual frameworks for different funding streams, with some duplication and inefficiency. As one interagency UN coordinator described:

In a very small country with a huge number of actors. . I think there is lot to learn and draw upon because things have evolved to, often, a very high technical specification. However, the coordination landscape. . then becomes incredibly messy because you’ve got so many different people. . different mandates, different global politics being played out at the country level.

Informants had varying perceptions of the effectiveness of Lebanon’s humanitarian architecture and agreed that the LCRP should have been adapted earlier to avoid establishing parallel response frameworks for COVID-19 and the Beirut Blast. Some suggested that the LCRP structure, which was initially designed to address the Syrian refugee crisis, should be restructured to reflect the nationwide economic and political crisis in Lebanon. They advocated for a more integrated and holistic approach to meet the expanding needs of both Lebanese and Syrian populations. As one UN coordinator described:

We cannot keep having things detached. . especially that Lebanon is no longer just the recipient of … [an] influx of people, but it’s a country of crisis. So, I would think that now it’s time to move to a different framework that includes the whole of Lebanon. . For the way forward, definitely to not have it led with the stamp of UNHCR/refugees… . now the needs are equally there. Lebanese and Syrians, they suffer the same.

However, implementing such changes would require repositioning the roles of UN organizations, including OCHA and UNHCR, and redistributing power across agencies. A proposal for a more comprehensive humanitarian response plan (HRP) with a stronger role for OCHA had been suggested due to the nationwide economic crisis. However, after two years of negotiations, the proposal was rejected, as the underlying causes of the crises were attributed to poor governance, requiring government reforms. Reaching consensus between different UN organizations was reportedly challenging due to differing agency interests, leading to a fragmented approach. As one UN coordinator put it:

We’ve been fighting about this for two years, we just need to get on and deliver. There wasn’t an agreement about an HRP, no one is pushing for that now, so let’s just try and focus on delivering the frameworks that we’ve got, and streamline coordination.

Despite the complex situation, many informants agreed that UNHCR should continue to play a leadership role under the LCRP while a significant refugee population still existed in Lebanon.

So basically, what we’re seeing in Lebanon is the competition between the new framework, which is the ERP, and the existing framework, which is the LCRP, UNHCR led and really focusing on refugees. Honestly, I would say that LCRP will remain the biggest response because you cannot just overlook the presence of 1.5 million refugees in Lebanon. . I don’t think that the framework will be anything else but refugees focused. Even if they try to push for a more integrated approach. (Government representative).

Importantly, coordination across all frameworks had been integrated at the sector level under the established LCRP interagency system. Throughout the various new crises the GBV task force remained the principal coordination mechanism for the GBV sub-sector in Lebanon by helping organisations work across, and take advantage of, opportunities in the new frameworks and adapt to the changing crisis context [7]. Informants emphasized that the pre-existing culture of trust and mutual solidarity, combined with positive interpersonal dynamics within the task force, facilitated successful responses, despite additional coordination challenges [59]. Notably, informants praised the rapid and dedicated response of national actors in the aftermath of the Beirut Blast, despite being personally impacted:

Beirut blast happened on a Tuesday. . and I remember on Thursday, we had our first meeting, noting that most of the people that were sitting in that meeting, helping coordinate, had their homes broken, shattered…We were that fast. (National GBV actor).

The GBV coordination task force was commended by informants for quickly adapting coordination to virtual mechanisms from the beginning of the COVID-19 pandemic. Monthly remote meetings with approximately 30 members, including UN, INGO, national and local organizations, researchers, and donors, were held throughout 2021-22. Nonetheless, the reduced in-person interactions hindered relationship building and informal coordination, vital for effective collaboration according to earlier studies [63]. Coordinators compensated for this with frequent emails, surveys, and bilateral communications to ensure broad engagement. The challenging economic and political context in Lebanon had resulted in higher staff turnover in recent years, leading to the departure of several long-term international GBV coordinators. Officially co-led by MOSA, UNHCR, and UNFPA, the UNHCR GBV coordinator’s position was vacant from 2019 to 2022. All of this created gaps in leadership, resulting in the loss of institutional and contextual knowledge and compromising the relationships built over the previous years.

Subnational coordination and the linkage between national and sub-national levels was highlighted by informants as crucial to enhancing service delivery at the field level. Sub-national coordinators in Lebanon praised the proactive and responsive approach of the national task force, which provided harmonized guidance and tools to support actors at the field level in adapting to remote modalities and responding to the COVID-19 pandemic.

The task force was actively looking for tools, adapting or developing tools trying to harmonize and provide guidance so that actors on the ground were not running around trying to figure out what the solution was, allowing them to respond in a safe and effective way ensuring the minimum standards were adhered to. There was a lot coming out from the task force during that period that really helped us to adapt to the pandemic. (UN Field coordinator).

Additionally, GBV actors implemented several mechanisms to strengthen the interface between national and sub-national coordination. This encompassed well-defined and complementary responsibilities, field coordinators participating in national meetings to understand GBV strategies, national coordinators joining sub-national meetings to convey priorities, and adapting guidelines and tools at the national level to ensure consistency across regions. National GBV coordinators also held six-weekly meetings with field coordinators, providing a valuable forum to share experiences and lessons across regions. This was highly valued, as one UN field coordinator described:

Super useful!… that’s what I find is the strength of this forum is it’s coordination, but it is also really a place to share experience, and share knowledge, and share lessons learned, and that’s how we’re building our expertise as a sector, because we have these spaces, and then when we can do that across regions, it even adds more value to that.

Moreover, intersectoral coordination involving Health, Protection, Gender, Shelter, Mental health and psychosocial support (MHPSS), Livelihoods, and other sectors proved pivotal in tackling multifaceted challenges stemming from the pandemic and economic emergencies. This collaborative approach enabled a more robust and holistic GBV response within the context of the compounded emergencies (Table 1). Informants acknowledged that the interagency system in place since 2013 played a crucial role in fostering intersectoral coordination and relationship building among sector coordinators, which also facilitated progress in GBV risk mitigation. GBV coordinators actively participated in CP, Protection, CMR, Gender, MHPSS and Health sector meetings, and co-led crosscutting initiatives such as Protection from Sexual Exploitation and Abuse (PSEA), all of which enabled integration of GBV and promoted cross-sector collaboration [9]. For example, rising mental health issues across all populations in Lebanon, coupled with a rise in substance abuse, heightened GBV risks, while the departure of skilled Lebanese workers, including doctors and psychiatrists, posed challenges for GBV survivors requiring specialized services like MHPSS. In 2023, the MHPSS, Protection, GBV, and CP working groups collaborated to conduct training for frontline workers, enhancing MHPSS identification and referrals. Additionally, rising GBV cases involving children and increasing child marriage, prompted improved protection sector coordination and enhanced programming for adolescent girls through joint GBV and CP coordination meetings. The LCRP also recognized GBV as a shared responsibility and mandated its integration as a key crosscutting issue across all sectors [13, 52].

### Coordination to support GBV service delivery

Despite having a robust GBV service network and a comprehensive referral system developed during the Syrian crisis response, the expansion of vulnerable populations brought new complexities and challenges to the delivery of services that the GBV task force had to overcome (Table 1) [9, 64]. GBV actors implemented a range of innovative approaches to adapt and sustain service delivery, ensuring that survivors’ needs were met despite the limitations posed by COVID-19 restrictions and shortages of electricity, fuel and internet connectivity in Lebanon. The remote modality was a barrier for some cases and informants commended the task forces at the national and sub-national levels for being creative and flexible to ensure service delivery continued and that high-risk cases could receive in-person services where possible. As described by one UN field coordinator:

We had the COVID 19 pandemic, intersecting with the fuel and electricity crisis, and so how can you provide remote services to individuals who can’t charge their phones, for example, because they don’t have electricity? So just dealing with those very practical, operational challenges in terms of creating a space, where, at the national and subnational level, we were really focusing a lot. . on sharing knowledge and experience. (UN Field coordinator).

At the same time, due to challenges with transportation and the fear of contracting COVID-19, some service users preferred remote modalities and several informants reported that engaging particular groups, such as members of the LGBTIQ + community, was enabled by the anonymity of remote engagement.

Additionally, informants credited an effective GBV information management system (GBVIMS), with a dedicated interagency coordinator, for supporting the GBV task force to assess the impact of the compounded crises and adapt GBV coordination and services accordingly. By 2023, GBVIMS was employed by 15 organizations to collect detailed GBV data in Lebanon, bolstering coordination with data to advocate for funding, develop policy briefs, and guide programming and service strategies. For instance, a 2020 assessment on the impact of COVID-19 on GBV identified an increase in domestic violence and decreased reporting of GBV incidents, highlighting the challenges faced by women during lockdowns as they were confined with perpetrators but could not report incidents or seek care [65]. Analysis of routine GBVIMS monitoring data enabled an understanding of the GBV impacts of COVID-19 on minority groups like LGBTIQ + communities. In addition, the National Commission for Lebanese Women (NCLW), partnered with UN stakeholders to issue gender and COVID-19 policy briefs throughout 2020-21 [66]. These provided recommendations for a more gender-equitable response and were disseminated across the humanitarian sector. In 2021, GBVIMS data showed an increase in the proportion of GBV cases reported among children from 9 to 13%, with forced marriage, psychological or emotional abuse and sexual assault most commonly reported [47]. In response, the GBVIMS steering committee issued a report with recommendations on safeguarding at-risk populations and responding to the needs of adolescent girls and child survivors [47, 67]. Efforts were also made to disaggregate GBVIMS data across diverse nationalities including Lebanese, Palestinian, and migrant populations, and to ensure that outreach and support efforts were inclusive of all nationalities.

Amid the national economic emergency and the growing size of vulnerable populations, informants emphasised that more funding flexibility was necessary to meet expanding needs. Currency devaluation and fluctuating exchange rates impacted service delivery costs, leading to a 20% increase in the GBV sub-sector funding request for 2022. The task force advocated for inclusion of GBV in Lebanon’s Humanitarian Fund and the ERP. The ERP, for example, allocated 3 million USD for three national NGOs and two international NGOs to implement service delivery, emergency protection cash, shelter aid, and GBV risk mitigation, though informants reported slow implementation as of May 2022. Frustrations about donor driven agendas and the lack of unrestricted and flexible funding, which especially limited local and national organizations, also became more acute during the economic crisis. Informants noted that donors and agencies allocated funding in line with their mandates and priorities, which were not always aligned with the needs on the ground and left some vulnerable populations without access to life-saving services, including the LGBTIQ + community, migrant workers, and Persons with Disabilities (PwDs). For example, local actors reported difficulties mobilizing funding to support GBV survivors in the LGBTIQ + community, Palestinian refugees, and female migrants. In response, the task force targeted efforts to mobilize funding to support these vulnerable populations to access to life-saving services. Agencies collaborated to create a policy brief on GBV in PwDs, emphasizing gaps and advocating for specialized interventions [68].

Both UN and local GBV actors shared the view that GBV prevention and women’s empowerment efforts were increasingly futile in the challenging context, jeopardizing long-term progress. They feared that the crisis would reverse the advancements made over the past decade and expressed concern that human rights were being overshadowed by the economic crisis. As one local GBV actor explained:

People used to access our centres and benefit from awareness sessions. They wanted to increase their knowledge about women’s rights, GBV, gender roles, how to have a more equal society, but this is all changing.

Women and girls became less able to participate in traditional GBV activities due to their inability to meet basic needs, and cash assistance became crucial. In response, UNFPA piloted integration of cash assistance within GBV case management services, positively impacting GBV risk mitigation and survivors’ access to comprehensive support. A 2022 report showed that cash assistance substantially reduced exposure to GBV incidents for 85% of service users and enabled or encouraged access to GBV response services for 82% [69]. However, the deteriorating economic situation and hyperinflation led to a reduction in the emergency cash transfer value, and the scale of assistance remained insufficient. Funding in dollars had to be provided in Lebanese pounds due to donor conditions, resulting in service users receiving only a fraction of expected assistance. As described by one local GBV actor:

… because of the devaluation of the lira what they [women] are receiving is peanuts … they call us and they say “you know you’re paid to give us money in dollars but what we get it’s not even covering the transport to come and pick it up and then go to the bank”.

While the pilot program offered cash assistance for up to six months, partners simultaneously worked with survivors on medium to long term livelihoods plans.

### Stakeholders’ roles, legitimacy and power

Political instability and perceived corruption in Lebanon’s government undermined its capacity to coordinate the response, eroding trust among donors who hesitated to provide direct funding and leading to exclusion of the government from the ERP. Furthermore, the government’s absence from the Beirut Blast response was attributed to weak disaster preparedness, while others blamed Hezbollah’s alleged involvement and their resistance to investigating the cause, eliciting strong criticism from our informants:

There’s no worse example than how the government responded to the blast. There’s no accountability, there’s no interest in what had happened, there is no commitment to the population. . if you can’t see beyond your petty sectarian issues for that, then you never will (National GBV actor).

GBV actors expressed frustration with the political system’s underlying patriarchy and corruption, believing that real change on GBV in Lebanon would require addressing these systemic root causes.

Our politicians all need to be taken out and dumped in the sea and [the system] overhauled completely. Because as long as we continue to recycle the same old men with the same old ideas, we are the classic example of a patriarchal [system] (National GBV actor).

Furthermore, the competing demands of the compounded crises meant that issues such as GBV, gender equality and PSEA were being deprioritized within government. As one government actor described:

When I tried to better mainstream PSEA in the work we do, this is the answer I’ve heard: “it’s really good. We want you to work on that and we want to talk to other ministers about it, but this is not the priority now. Our priority is focusing on the COVID response, and. . [those] affected by the economic crisis”.

Despite the challenges posed by an unstable government, corruption, and weakened legitimacy, both UN and government actors emphasized the importance of maintaining government-co-led interagency coordination of the humanitarian response under the LCRP. The dedicated interagency system facilitated engagement with government actors on GBV and protection, and alongside dedicated funding, allowed humanitarian actors to influence government refugee policies. This was considered critical to ensure protection for refugees in Lebanon, as described by one UN coordinator:

Because I personally am convinced on this point and I think it does underpin the whole raison d’être of the LCRP, that by maintaining this co-led response, this is one of the keyways in which we are protecting the protection space for refugees here. . that’s the space that we actually use to negotiate the decisions that are being made and try to prevent harm in terms of governmental policy decisions that would be harmful to refugees.

Furthermore, excluding the government from coordination and decision-making, as was the case with the ERP, was criticized by government actors, who argued that it would compromise the sustainability and impact of the assistance being delivered under the LCRP:

You cannot marginalize the government when working in a country. First of all, you’re working under their authority and you’re working under their jurisdiction. Secondly, if you’re working as a humanitarian actor, to ensure sustainability, because you’re not going to stay here forever, you need to ensure coordination with the government. So even if you don’t want to channel funds through the government you still need to coordinate and engage the government in decision making. . And I think marginalizing governments is a bad practice and will never help with sustainability and with the efficiency of the programs. (Government representative).

By co-leading the GBV task force that functioned across all of the emergency response frameworks, the government retained its involvement in key coordination discussions and decision-making processes. Since 2020, for example, MOSA’s LCRP protection coordinator played a leading role in GBV task force meetings, serving as a liaison between GBV actors and MOSA. They facilitated government review and approval of key decisions and documents while providing government perspectives during GBV task force discussions. Informants acknowledged the importance of government actors’ contextual knowledge and experience, particularly given the high turnover of humanitarian actors, reiterating the importance of bolstering government leadership [52].

Nevertheless, there was prevailing cynicism among informants about the government’s willingness, commitment and capabilities to assume responsibilities in GBV service provision, captured succinctly in one local actor’s response: *“Maybe in another life”.* At the local level, progress made throughout the Syrian crisis on institutionalizing GBV services within government-run social development centers (SDCs), was being threatened due to the challenging operating context. SDCs, supported by UN, INGOs and local actors, provided information, support, and referral for GBV services to women and girls [64]. In recent years, however, SDCs were operating at a much-reduced capacity, because of challenges related to government staff payment and a lack of fuel and power. As described by one local actor:

They make an effort to be involved and they try to be present, but because sometimes they go two, three months without pay. And now with a market fluctuation, they get paid in Lebanese pound, which is basically nothing in comparison to the prices at the market. So, you feel that they are no longer devoted, they’re not really convinced that they can make a difference. (Local GBV actor).

CMR services at public hospitals were also being compromised. For example, Lebanon Rafiq Hararri Hospital in Beirut, which was an important a hub for vulnerable populations to access free medical services, was designated as a COVID-19 treatment facility and not operating at full capacity, leaving a major gap in CMR service provision.

Consequently, since 2019, the role of national and local NGOs had become increasingly important but evermore challenging. Highly influential within the GBV task force at both national and sub-national level, informants deemed it crucial to empower national NGOs to take on GBV coordination leadership roles. As one local GBV actor explained:

We need to make sure that local NGOs are also in the leadership positions, not only supporting in the decision making,. . and make sure that we really have those strong feminist organizations at the forefront.

Their trusting relationships with, and access to, communities, which was important for programming on a sensitive issue like GBV, became increasingly important during Lebanon’s compounded crisis period as new challenges arose. For example, in response to the rise in child marriages, especially pronounced in the north of the country, GBV actors intensified efforts to reach adolescent girls through remote support to outreach volunteers in Akkar and Bekaa. In addition, several agencies engaged religious leaders to prevent child marriage, a guidance note on child GBV survivor care was developed and a decision tree created for referring child GBV survivors to case management services. More broadly, through capacity building efforts over the past decade, a gradual evolution had occurred to a semi-sustainable system, whereby UN agencies directly funded national and local NGOs to define GBV programming needs, solutions and provide GBV services [7]. While this strengthened sustainable local capacity, the system remained reliant on international funding. The majority of national and local actors commended the significant and successful investments made by UN and INGOs since 2012, however, some questioned the UN’s commitment to completely transition service implementation to local actors, suggesting it might be a way to maintain control of funding.

Well, you guys have been building our capacities for the last five years. So, if we don’t know how to do this, that’s shame on you. . your aim is to transition to local NGO, then local NGO to transition to governmental entities. That’s why we’re working in close coordination with the SDCs of MOSA. (Local GBV actor).

During Lebanon’s multiple crises, national and local actors were directly affected, and the GBV task force recognized the importance of supporting staff well-being. As one local actor described: *“The economic crisis has just destroyed everybody’s lives.”* High rates of staff burnout were reported, making it challenging to sustain GBV service provision. The GBV task force were commended across the humanitarian sector for assessing the impact of the situation on frontline workers and highlighting this with donors and other stakeholders. Results of an assessment conducted by the GBV task force in August 2021 showed that 27% of providers reported a severe impact on their capacity to deliver services, while 96% reported difficulties for users to access GBV services [36]. Local actors implemented measures to ensure service continuity, which included augmenting support for communication and transportation expenses for both survivors of GBV and staff members. In addition, staff safety concerns were high, with 94% of service providers indicating an impact on safety, and some reporting incidents of thefts and threats. Several organizations were able to provide psychosocial support to staff but 18% lacked funding for such services [36]. The GBV task force highlighted the importance of prioritizing and funding the GBV sector during the 2021 16 Days of Activism, given rising GBV risks, growing vulnerable populations, and added pressures on local actors amidst the compounded crises.

## Discussion

Since 2019, the continued socioeconomic decline in Lebanon, amplified by compounded crises and government inaction, have heightened vulnerability of refugees and Lebanese populations alike, elevating GBV risks. Humanitarian response has been repeatedly reformulated to adapt to the multiple crises, which have complicated GBV coordination and service delivery, and which have required flexible approaches. Our findings underscore the importance of responsive and adaptable GBV coordination to enable GBV actors to continue providing essential services to survivors while navigating the complexities and additional needs posed by the compounded crises. Lebanon offered a unique setting to examine GBV coordination in complex emergencies and below we discuss important lessons applicable to GBV coordination in both Lebanon and other complex emergencies.

Experience from Lebanon underscores the need for coordination systems to suit the context while being flexible to adapt and evolve as the context changes [4, 59, 70, 71]. Although the interagency coordination system established under the LCRP in Lebanon had matured throughout the Syrian crisis, with international humanitarian actors increasingly working in partnership with government and national organizations, the compounded crises since 2019 have challenged this system to further evolve [59]. Diverse organizational agendas and political instability resulted in a complex architecture of response frameworks that stakeholders had to navigate [15, 72, 73]. Constructively, coordination was streamlined and integrated within the established LCRP interagency coordination and the GBV task force remained the principal coordination mechanism for GBV actors in Lebanon. The GBV task force benefited from mutual solidarity and trust built during the response to the protracted Syrian crisis, but faced challenges due to remote working and high levels of coordinator turnover. Effective sub-national GBV coordination and inter-sectoral coordination were crucial in addressing GBV during the compounded emergencies in Lebanon, as noted in other settings too [9, 11, 59, 74]. The strong interface between national and sub-national structures enhanced the overall system’s strength, ensuring a harmonized GBV approach across the country, while enabling different regions to tailor coordination and services to their unique local contexts. The strong linkage also facilitated the exchange of lessons and experiences across regions. Approaches implemented in Lebanon to strengthen the interface could be replicated in other crisis-affected countries. Periodic review of the structure and roles of the humanitarian architecture is necessary to ensure that GBV coordination accounts for the evolving response context, in line with global recommendations [59]. Continued investment in building a culture of trust, solidarity, and inclusiveness in GBV coordination in Lebanon and other complex contexts with limited resources, is recommended [28, 75].

Globally, the COVID-19 pandemic posed major challenges for GBV service delivery and in Lebanon this was further compounded by the severe economic crisis and the devastation caused by the Beirut Blast. While comprehensive and specialized GBV programs remain essential during public health crises, access to such services globally, including in Lebanon, reduced, as health services were repurposed to respond to the pandemic, a trend also noted in other public health emergencies [11, 43, 76–78]. The GBV task force developed contextualized guidance, adapted services, and revised referral pathways to ensure GBV services were available to survivors, and promoted a harmonised response across the country, aligning with global recommendations [79, 80]. The task force paid particular attention to vulnerable populations, including the LGBTIQ + community, female migrants, PwDs and adolescent girls, and developed specific recommendations to safeguard their needs and respond to their unique challenges during the compounded crises period [68]. Additionally, GBVIMS played a critical role in providing data to assess and adapt services and coordination. While remote service provision presented challenges, it also fostered innovation that benefited some marginalized populations, such as LGBTIQ + individuals. Some of these innovations could be sustained or expanded post COVID-19. While GBV in public health emergencies has traditionally been neglected, we have seen major improvements throughout the COVID-19 pandemic, both globally and in Lebanon [78, 81]. New guidelines, coordination strategies and programming approaches developed and piloted during the COVID-19 pandemic, including those identified by this study, should be consolidated and global lessons integrated into future responses [82].

Lebanon’s GBV response relies heavily on international funding, and increased predictable, multi-year funding is necessary due to rising GBV risks posed by the compounded crises, currency devaluation, and increasing operational costs [44. Furthermore, fragmented response frameworks, donor driven agendas and strict organizational mandates left some populations without lifesaving GBV services [9]. Moving away from donor driven agendas, which our findings noted are unaligned with the needs on the ground, and providing non-earmarked, flexible funding, would allow organizations to adapt to an extremely volatile context [36]. This reinforces previous findings, which suggest that in rapidly evolving contexts, needs frequently change and funding must adapt accordingly [50, 72]. Humanitarian actors should improve monitoring to track GBV-specific investments, and disaggregate funding allocations across GBV response, risk mitigation and prevention programming, which is currently lacking in the LCRP [82–85]. Furthermore, GBV prevention, including women’s economic empowerment, requires long term investment as the multiple layers of crisis in Lebanon are threatening progress, which warrants intensified funding and attention [9, 83].

In recent years, cash assistance has provided much needed support for GBV survivors in Lebanon and other humanitarian settings [86]. However, the impact of cash programmes in Lebanon was undermined by the scale of the economic crisis [69, 87]. Furthermore, refugees’ right to work in Lebanon is severely restricted, often resulting in deepening poverty and worsening harmful coping strategies like child marriage and survival sex [64]. Cash assistance can empower GBV survivors economically, reduce dependency on perpetrators, and address underlying economic risk factors contributing to GBV and has shown positive impacts in some settings [86]. However, its success hinges on careful program design, integration with comprehensive GBV services, safety considerations, and context-specific approaches and more research is needed to establish clear evidence [86]. Based on preliminary findings in Lebanon, the GBV task force should expand longer-term cash assistance, taking into account rising inflation and the dollarization of services to better serve survivors, which may also help to mitigate the risks of harmful coping mechanisms [69, 77]. More broadly, targeted integration efforts are required to expand Syrian refugees’ access to services and right to live, move and work, including through refugee-led initiatives in humanitarian and development programmes. Given that refugees constitute a significant portion of Lebanon’s population, harnessing their human resource potential is vital to assist Lebanon in overcoming the effects of its compounded crises.

A perhaps obvious but nevertheless important point emphasised by previous research, and reaffirmed by this study, is the key role that national governments play in leading and coordinating humanitarian assistance in a country [59, 88, 89]. The volatile context and government instability cast doubts over the Lebanese government’s legitimacy, willingness and capability to coordinate and sustain GBV work, mirroring issues noted in other settings [70, 90, 91). Nevertheless, our findings underscore the importance of maintaining a government co-led humanitarian response (28, 92]. Although humanitarian assistance can alleviate suffering, it is insufficient to tackle the underlying causes of complex emergencies like the situation in Lebanon. Addressing this requires governance and institutional reforms alongside regional and global solutions to facilitate Lebanon’s recovery [21, 28, 56].

A weak government, erosion of state institutions and the exodus of high-skilled professionals have made the role of national and local women’s organisations in sustaining GBV services and coordination ever more important, underscoring the need to advance localization. Although global GBV localisation targets have not been met, investments in Lebanon throughout the Syrian crisis have demonstrated some positive impact [11, 93]. Lebanon’s experience highlights the value of investing in partnerships and enhancing local capacity, as these entities maintained service provision amid the compounded crises, facilitated by robust coordination at both the national and sub-national levels. Growing refugee-host tensions, security incidents, movement restrictions, and access barriers during COVID-19 reinforce the importance of building sustainable local capacity [28, 94–97]. Additionally, the challenges faced by frequent turnover of international GBV coordinators present the potential to transfer coordination responsibilities to national and local organizations, particularly in protracted emergencies [70]. Furthermore, investing in national and local organizations will become increasingly important for Lebanon’s recovery from compounded crises, especially when donors restrict funding to the government [21, 28, 36]. In 2023, UNDP mapped women-led organizations (WLOs) in Lebanon and analysed their role in advancing effective humanitarian action [98]. The report recommended improved coordination via access to decision-making fora, integration, language adaptation, and funding, stressing the recognition of their existing capacities. Subsequently, national GBV coordinators introduced the GBV task force structure to WLOs, and sub-national coordinators invited them to join coordination mechanisms, underscoring their significance for GBV coordination in the complex and dynamic context [98]. Prioritizing the inclusion of refugee-led organizations in coordination is also crucial, drawing insights from contexts with successful localization, such as Syrian cross-border operations in Türkiye [93, 99]. Finally, we argue that the compounded crises could be leveraged to promote gender equality by ensuring that women are in leadership positions in organisations involved in Lebanon’s recovery. Women’s organizations should be funded and supported to engage at all levels of leadership and decision-making as a positive force in Lebanon’s recovery [100].

### Limitations

Due to COVID-19 pandemic restrictions, data collection was conducted remotely, which limited engagement with civil society actors, donors and government actors. The range of informants interviewed for this study is limited and does not reflect the full spectrum of humanitarian actors involved in the response. Service users were not interviewed as part of this research but could have provided important insights on the ways in which coordination challenges and initiatives are experienced by people targeted by GBV programming.

## Conclusion

To the best of our knowledge, this study represents the first exploration of GBV coordination within a complex compounded crisis, offering insights to enhance GBV coordination across similar contexts. Our findings highlight the essential role of government leadership in coordination; the value of investing in local capacity-building during chronic crises, which pays off when crises expand or become compounded; the significance of effective national and subnational coordination and a harmonized national approach for supporting service delivery; the importance of strong intersectoral coordination to address cross-cutting GBV issues during public health and complex emergencies and; the need to prioritize the well-being of front-line staff in all types of crises. In addition, our findings underscore the necessity of targeted programming for vulnerable populations, such as adolescent girls, PwDs and LGBTIQ + individuals, in Lebanon and similar contexts [77]. Donors are encouraged to increase flexible, multiyear funding for GBV coordination and services in such crises, to allow organizations to adapt to volatile contexts. In complex emergencies, periodic review of the structure and roles of the humanitarian architecture is necessary to ensure that GBV coordination accounts for the evolving response context and that women-led organizations are at the forefront of recovery efforts, contributing to more equitable societies [28].

### Electronic supplementary material

Below is the link to the electronic supplementary material.

Additional File 1: Qualitative Interview Guides used for semi-structured interviews.

Additional File 2: Table 2: Documents included in the analysis.

## Data Availability

The datasets analysed during the current study are available from the corresponding author on reasonable request.

## Abbreviations

CMR: Clinical Management of Rape
COVID-19: Coronavirus-19
CP: Child Protection
CSO: Civil Society Organization’s
ERP: Emergency response plan
GBV: Gender Based Violence
GBVIMS: Gender Based Violence Information Management System
INGO: International non-governmental organization
LCRP: Lebanon Country Response Plan
LGBTIQ+: Lesbian, gay, bisexual, transgender, intersex, queer, +
MHPSS: Mental health and psychosocial support
MOSA: Ministry of Social Affairs
NGO: Non-governmental organization
PSEA: Protection from Sexual Exploitation and Abuse
PwD: Persons with Disabilities
SDC: Social Development Centre
UN: United Nations
UNDP: United Nations Development Programme
UNFPA: United Nations Population Fund
UNHCR: United Nations High Commissioner for Refugees
UNICEF: United Nations Children’s Fund
UN OCHA: United Nations Office for the Coordination of Humanitarian Affairs
WHO: World Health Organization
