# Supplementary material for: Gender based violence (GBV) coordination in a complex, multi-crisis context: a qualitative case study of Lebanon’s compounded crises (2019–2023)
Source: Confl Health. 2023 Oct 23;17:50. doi: 10.1186/s13031-023-00543-8 (PMC10591352; doi:10.1186/s13031-023-00543-8)
Supplement: Supplementary file 1 — Additional File 1: Qualitative Interview Guides used for semi-structured interviews. [file 13031_2023_543_MOESM1_ESM.docx]

## Additional File 1: Topic Guides for In-depth Interviews

**Qualitative Interview Guides**

**Objective: To gather information on the evolution of the Gender-based violence (GBV) coordination system in Lebanon’s humanitarian response**

The interview topic guides are structured as follows:

1. Individuals involved in development of the GBV Coordination Mechanism since 2011 - Evolution of the GBV Coordination mechanism
2. Individuals currently involved in GBV Coordination – UN and iNGO Humanitarian actors, Government, National NGO’s, Research Institutes
3. Government and Humanitarian Actors in other sectors of the Response
4. National and Local Actors working on GBV and Gender issues

**Topic Guide 1: Individuals involved in Development of the GBV Coordination Mechanism since 2012**

**Evolution of the GBV Coordination mechanism**

I would like to start by asking you a few questions about how the GBV coordination system works in Lebanon.

1. Briefly describe your role and responsibilities within the GBV Coordination mechanism.
2. Can you describe the GBV coordination mechanism for the humanitarian response in Lebanon? Who are the main agencies and actors?
   1. Prompt: National and Field level, Inter-sectorial coordination, role of National Government, Key actors and stakeholders
   2. How was it initially set up and how has it changed over the course of the protracted crises?
3. What are the different levels of coordination – National, Field level and what are the different roles and responsibilities at each level?
   1. Who are the key players at each level?
4. Can you identify any key milestones and events which significantly influenced how the GBV coordination mechanism developed in Lebanon?
5. Can you think of any innovative solutions that were created to support GBV coordination?
6. How does this GBV coordination mechanism compare with or differ from those in other contexts where you have worked?
   - - 1. Prompt: Urban response, Protracted crisis, no camp policies
7. Do you have any examples of policy decisions which influenced the GBV coordination mechanism over the course of the response? Prompt: Call to Action, Repeal of rape law and Grand Bargain

**Context – Stakeholders**

1. Who are the actors (local, national and international) and stakeholders working on GBV and involved in the coordination mechanism in Lebanon
   1. Can you identify “GBV champions” over the course of the response?
2. Who have been key leaders in the GBV response? How have they influenced the evolution of the GBV coordination mechanism in Lebanon? (national, international levels)
   1. What has been the role of the National Government in GBV humanitarian response in particular?
3. Who are the key external stakeholders? How does the GBV coordination mechanism interact with the Broader humanitarian response actors & other sectors and National Government
4. What, if any, do you see as external factors which may impact the success of the GBV coordination system?
   1. Prompt: Political will, Government and Humanitarian Policies, Donor priorities, Access to resources

**Localisation**

1. What is the role of National NGO’s, Local Actors and civil society in the GBV coordination mechanism in Lebanon?
2. What, if any, is the role of refugee committees and women’s movement groups in the GBV coordination mechanism in Lebanon?
3. How were local organizations engaged in planning to ensure existing local strategies were strengthened and not undermined.
4. Were local organizations involved in assessments and needs/gap analysis and priority setting?
5. Working with local actors are they real partnerships or just cosmetic for funding purposes?
6. How do you support and build capacity of these local organisations?
7. What assessments were performed at early stages – was GBV considered in multi sector rapid needs assessments?
   1. Were local organizations involved in assessments and needs/gap analysis and priority setting?

**Field-level Implementation - Tracer Services**

1. What services were offered at beginning and how did that change over time?
2. Who is implementing which services? Names of organisations.
3. Was service mapping performed in early stages and regularly updated?
4. At what stage were SOP’s and referral pathways developed?
5. How does or does not GBV coordination translate into field-level implementation?

**Prioritization**

1. Can you describe how the GBV response has been prioritized and mainstreamed within the Humanitarian response in Lebanon?
   1. What factors do you believe influenced how each humanitarian sector prioritised GBV response?
2. Describe the national GBV prevention and response policy context in Lebanon’s humanitarian response?
   1. How do you think this influences, if at all, how GBV is prioritized and addressed in the Humanitarian response in Lebanon?
3. Describe the cultural context concerning GBV prevention and response in Lebanon at the national level. How do you think this may influence how GBV is prioritized and addressed in the humanitarian response?
   - 1. Prompt: cultural practices, social norms, harmful gender practices, human rights, women’s rights, child rights

**Integration and Mainstreaming**

1. What are the key sectors the GBV coordinators work with? What does this work involve?
2. What influences how is GBV integrated and mainstreamed by other sectors?
3. How does the context - political discourse, policies, social and cultural norms - influence integration of GBV in the humanitarian coordination system in Lebanon?
   1. Please tell me about the roll-out of the 2015 GBV Guidelines. Has this affected how GBV is addressed in humanitarian response? If yes, how?
4. What have been barriers, if any, to GBV integration into humanitarian coordination system to date?
   1. How can these been overcome?

**Political unrest and Beirut blast**

1. How has the recent political unrest in Lebanon and the Beirut blast impacted your work?
2. How are coordination mechanisms being adapted in response to the emergencies?
   1. Has this negatively affected your work?

**COVID-19 Pandemic**

1. How has the covid pandemic impacted your work?
2. How are coordination mechanisms being adapted in response to the pandemic?
   1. Has this negatively affected your work?

**Future Direction**

1. Do you believe sustainability is being addressed in relation to the continuation of GBV programmes and coordination in the protracted crisis?
2. How do you think sustainability of GBV programs and coordination structures can be built in the protracted crisis?
   1. Probe: Resource allocation, Donor prioritization, Partnership with Local Actors
3. What if anything needs to change with the current system to ensure sustainability in the protracted crisis?
   1. What if anything already has changed?

**Conclusion**

1. What are the strengths, if any, of the existing GBV coordination model in the current protracted crisis?
2. What could improve the existing GBV coordination mechanism in Lebanon?
3. What important lessons could we learn and apply to other contexts?
4. Do you have anything else to add? Suggestions? Recommendations?
5. Are there other individuals or organizations you can suggest I speak with regarding the GBV coordination mechanism in Lebanon?

**Topic Guide 2: Those currently involved in GBV Coordination – UN and iNGO Humanitarian actors, Government, National NGO’s, Research Institutes**

I would like to start by asking you a few questions about how the GBV coordination system works in Lebanon.

1. Briefly describe your role and responsibilities within the GBV Coordination mechanism.

**Context – Stakeholders, Partnerships and Policy Context**

1. Can you describe the GBV coordination mechanism for the humanitarian response in Lebanon? Who are the main agencies and actors?
2. What are the different levels of coordination – National, Field level and what are the different roles and responsibilities at each level?
   1. Who are the key players at each level?
3. Describe the GBV Policy Context in relation to the GBV coordination system in Lebanon

Prompt: Key policies, guidelines, frameworks, Political will

1. What has been the role of the National Government in the response? In GBV in particular?
2. What is the role of each of the following the GBV coordination mechanism in Lebanon?
   1. National NGOs
   2. Local Actors
   3. Civil society
   4. community-based organisations
   5. refugee groups or women’s groups
   6. political movement groups
3. What challenges does your organisation experience operating in this context?

Prompt: Urban context, protracted crisis, no-clusters

1. Would you describe the environment as enabling for innovation for GBV response?
2. What do you see as the external factors which may impact (barriers/enablers) the success of the GBV coordination?
3. How would you describe the leadership style used within the GBV Coordination mechanism?
   1. How do you think this influences the success of the coordination?
   2. How are priorities agreed?
   3. How are decisions made on GBV prioritization and integration?
   4. How are collective outcomes agreed and how often is strategic planning updated?
4. How does the GBV coordination mechanism in Lebanon compare or differ from other contexts in which you have worked?

**Resources and information**

1. Are GBV coordination resources sufficient in this protracted crisis?
   1. Funding?
   2. Technical resources?
   3. Human resources?
   4. Time?
2. Briefly describe how resources are mobilized and distributed within the GBV coordination system
3. What and how is GBV information is generated, managed and communicated? How?
4. How is information and evidence used to inform the functioning GBV coordination system and prioritization and integration of GBV programs?
5. Would you describe the flow of GBV coordination communication as bottom-up or top-down?

**Localisation**

1. What is the role of National NGO’s, Local Actors and civil society in the GBV coordination mechanism in Lebanon?
2. What, if any, is the role of refugee committees and women’s movement groups in the GBV coordination mechanism in Lebanon?
3. How are local organizations engaged in planning to ensure existing local strategies were strengthened and not undermined.
4. Are local organizations involved in assessments and needs/gap analysis and priority setting?
5. Working with local actors are they real partnerships or just for funding purposes?
6. How do you support and build capacity of these local organisations?

**Field-level Implementation - Tracer Services**

1. What services are available at field level?
2. Who is implementing which services? Names of organisations.
3. How does or does not GBV coordination translate into field-level implementation?
4. What, if any, results of GBV coordination in field level implementation are you aware of?
   1. If yes, are there relevant reports or other documents you could share with me?

**Prioritization and Integration**

1. Would you say that GBV is prioritized within the broader humanitarian response and what factors influence this?
2. Who sets the GBV priorities within the GBV coordination mechanism and the wider Humanitarian response?
3. What strategies are being used to integrate and mainstream GBV within the Humanitarian response
   1. What frameworks and guidance do you use for integration of GBV and how are humanitarian actors held accountable?
   2. Please tell me about the roll-out of the 2015 GBV Guidelines. Has this affected how GBV is addressed in humanitarian response? If yes, how?

**Behaviours**

1. How would you describe the values and culture within the GBV coordination mechanism?

Prompt: Trust, shared values

1. How would you describe how colleagues within the GBV coordination mechanism interact with one another?
   1. How would you describe the organizational culture of your organization?
   2. How would you say this fits with the culture of the GBV Coordination mechanism?
   3. In your role, do you every experience tension between competing priorities of the GBV Coordination mechanism and your own organization?
   4. How do you think this may or may not influence GBV coordination?
2. How would you describe the range of skills and competencies within the GBV Coordination mechanism?
   1. Are they adequate for the needs?

**Political unrest and Beirut blast**

1. How has the recent political unrest in Lebanon and the Beirut blast impacted your work?
2. How are coordination mechanisms being adapted in response to the emergencies?
   1. Has this negatively affected your work?

**COVID-19 Pandemic**

1. How has the covid pandemic impacted your work?
2. How are coordination mechanisms being adapted in response to the pandemic?
   1. Has this negatively affected your work?

**Future Direction**

1. Do you believe sustainability is being addressed in relation to the continuation of GBV programmes and coordination in the protracted crisis?
2. How do you think sustainability of GBV programs and coordination structures can be built in the protracted crisis?

Probe: Resource allocation, Donor prioritization, Partnership with Local Actors

1. What if anything needs to change with the current system to ensure sustainability in the protracted crisis?
   1. What if anything already has changed?

**Conclusion**

1. What are the strengths, if any, of the existing GBV coordination model in the current protracted crisis?
2. What could improve the existing GBV coordination mechanism in Lebanon?
3. What important lessons could we learn and apply to other contexts?
4. Do you have anything else to add? Suggestions? Recommendations?
5. Are there other individuals or organizations you can suggest I speak with regarding the GBV coordination mechanism in Lebanon?

**Topic Guide 3: Government, Donors. and Humanitarian Actors in other sectors of the Response**

I would like to start by asking you a few questions about how the Humanitarian coordination system works in Lebanon.

1. Briefly describe your role and responsibilities within the Humanitarian Coordination mechanism.
   1. How long has your organization been working on the response in Lebanon?
   2. Briefly describe how your work has changed over the course of the response?
2. Can you describe the coordination mechanism for the humanitarian response in Lebanon? Who are the main agencies and actors?
3. What are the roles of the different organisations? National and Field level, Inter-sectorial coordination, role of National Government, Key actors and stakeholders
4. Briefly describe the governance structure of the Humanitarian response. How does GBV fit within this governance structure?

**Prioritization and resources**

1. Do you participate in the humanitarian coordination mechanism in Lebanon?
   1. When was the last time you attended a meeting?
2. Briefly describe your role and your involvement with the GBV coordination system.
   1. Describe your organisations relationship or involvement with the GBV coordination system.
3. How does your organization consider and address GBV as a priority in the response
   1. If not why not?
   2. If yes, please provide examples to demonstrate this
   3. What frameworks and guidance do you use for integration of GBV and how are humanitarian actors held accountable?
4. How does the GBV coordination mechanism in Lebanon compare or differ from other contexts in which you have worked?
5. Is GBV adequately funded compared with the overall response?
   1. How could this be improved?

**Integration and Mainstreaming**

1. What do you see as the external factors which may impact the success of the GBV coordination system in Lebanon?
   1. Probe: political, policy, access to resources
2. What are the contextual challenges of addressing GBV in the humanitarian response in Lebanon?
   1. Probe: Urban setting, protracted crisis

**Localisation**

1. What is the role of National NGO’s, Local Actors and civil society in the GBV coordination mechanism in Lebanon?
2. Are local organizations engaged in planning, assessments and needs/gap analysis and priority setting?
3. When working with local actors are they real partnerships or just cosmetic for funding purposes?
4. How do you support and build capacity of these local organisations?

**Field-level Implementation - Tracer Services**

1. What services does your organisation provide?
2. Do you think coordination translates into results at field level for GBV response?
   1. If yes, do you have any examples you could tell me about?
   2. If no, why not?
3. What could be improved in terms of support to improve and sustain the delivery of integrated GBV services at the field level?

**Political unrest and Beirut blast**

1. How has the recent political unrest in Lebanon and the Beirut blast impacted your work?
2. How are coordination mechanisms being adapted in response to the emergencies?
   1. Has this negatively affected your work?

**COVID-19 Pandemic**

1. How has the covid pandemic impacted your work?
2. How are coordination mechanisms being adapted in response to the pandemic?
   1. Has this negatively affected your work?

**Future Direction**

1. Do you believe sustainability is being addressed in relation to the continuation of GBV programmes and coordination in the protracted crisis?
2. How do you think sustainability of GBV programs and coordination structures can be built in the protracted crisis?
   1. Probe: Resource allocation, Donor prioritization, Partnership with Local Actors
3. What if anything needs to change with the current system to ensure sustainability in the protracted crisis?
   1. What if anything already has changed?

**Conclusion**

1. What are the strengths, if any, of the existing GBV coordination model in the current protracted crisis?
2. What could improve the existing GBV coordination mechanism in Lebanon?
3. What important lessons could we learn and apply to other contexts?
4. Do you have anything else to add? Suggestions? Recommendations?
5. Are there other individuals or organizations you can suggest I speak with regarding the GBV coordination mechanism in Lebanon?

**Topic Guide 4: National and Local Actors working on GBV and Gender issues in Lebanon**

I would like to start by asking you a few questions about your work and how the GBV coordination system works in Lebanon.

1. Briefly describe your work and role in supporting the GBV and humanitarian response in Lebanon.
2. How long has your organization been working on the response in Lebanon?
3. Briefly describe how your work has evolved over the course of the response?
   1. Probe: Urban setting, protracted crisis

**Context – Stakeholders, Partnerships and Policy Context**

1. What support , if any, does your organization receive from the GBV coordination system?
2. What, if any, support do you receive in your role, from the GBV coordination system?
   1. Probe: Trainings, meetings, resources (technical, financial, human, logistical, operational, time)
3. If you receive support in your role from the GBV coordination system, what do you think of that support?
   - 1. Probe: positive versus negative aspects of that support
4. Have you partnered with any International agency or received funding for your work on GBV response?
   1. If so, in your opinion was this a real partnership or more cosmetic for funding purposes?
   2. How could this be improved?
5. What do you see as the external factors which may impact the success of the GBV coordination system in Lebanon?
   1. Probe: political, policy, access to resources
6. What are the contextual challenges of addressing GBV in the humanitarian response in Lebanon?
   1. Probe: Urban setting, protracted crisis
7. Do you work with the government, if so how do you find that relationship?

**Localisation**

1. What is the role of National NGO’s, Local Actors and civil society in the GBV coordination mechanism in Lebanon?
2. What, if any, is the role of refugee committees and women’s movement groups in the GBV coordination mechanism in Lebanon?
3. Are local organizations engaged in planning, assessments and needs/gap analysis and priority setting?
4. When working with local actors are they real partnerships or just for funding purposes?
5. How do international actors support and build capacity of these local organisations?

**Field-level Implementation - Tracer Services**

1. Do you think coordination translates into results at field level for GBV response?
   1. If yes, do you have any examples you could tell me about?
   2. If no, why not?
2. What could be improved in terms of support to improve and sustain the delivery of integrated GBV services at the field level?

**Prioritization**

1. Do you participate in the humanitarian coordination mechanism in Lebanon?
   1. When was the last time you attended a meeting?
2. Briefly describe your role and your involvement with the GBV coordination system.
   1. Describe your organisations relationship or involvement with the GBV coordination system.
3. How does your organization consider and address GBV as a priority in the response

**Political unrest and Beirut blast**

1. How has the recent political unrest in Lebanon and the Beirut blast impacted your work?
2. How are coordination mechanisms being adapted in response to the emergencies?
   1. Has this negatively affected your work?

**COVID-19 Pandemic**

1. How has the covid pandemic impacted your work?
2. How are coordination mechanisms being adapted in response to the pandemic?
   1. Has this negatively affected your work?

**Future Direction**

1. Do you believe sustainability is being addressed in relation to the continuation of GBV programmes and coordination in the protracted crisis?
2. How do you think sustainability of GBV programs and coordination structures can be built in the protracted crisis?
   1. Probe: Resource allocation, Donor prioritization, Partnership with Local Actors
3. What if anything needs to change with the current system to ensure sustainability in the protracted crisis?
   1. What if anything already has changed?

**Conclusion**

1. What are the strengths, if any, of the existing GBV coordination model in the current protracted crisis?
2. What could improve the existing GBV coordination mechanism in Lebanon?
3. What important lessons could we learn and apply to other contexts?
4. Do you have anything else to add? Suggestions? Recommendations?
5. Are there other individuals or organizations you can suggest I speak with regarding the GBV coordination mechanism in Lebanon?

**Topic Guide 5: Those involved in Global. And Regional GBV Coordination**

I would like to start by asking you a few questions about how the GBV coordination system works in the Region.

1. Briefly describe your role and responsibilities within the GBV Coordination mechanism.
2. Can you describe the GBV coordination mechanism for the humanitarian response in the MENA? Who are the main agencies and actors?
   1. How was it initially set up and how has it changed over the course of the protracted crises?
   2. Regional Coordination for WoS
3. What are the different levels of coordination – National, Field level and what are the different roles and responsibilities at each level?
   1. Who are the key players at each level?
4. How does each level coordinate and communicated with the other?
5. As Regional coordinator, do you work with Lebanon at all?
   1. What does this work involve?
   2. What impact do you think this has at a local level?
6. How does this GBV coordination mechanism compare with or differ from those in other contexts where you have worked?
   - 1. Prompt: Urban response, Protracted crisis, no camp policies
7. What has been the role of the National Government in GBV humanitarian response in particular?
8. What is the role of National NGO’s, Local Actors and civil society in the GBV coordination mechanism?
9. Describe the impact of Global GBV initiatives such as the call to action and New way of working on country level
   1. Do you have any examples of policy decisions which influenced the GBV coordination mechanism over the course of the response? Prompt: Call to Action and Grand Bargain

**Prioritization and Integration**

1. Would you say that GBV is prioritized within the broader humanitarian response and what factors influence this?
   1. Prompt: Humanitarian organizational cultures
2. What strategies are being used to integrate and mainstream GBV within the Humanitarian response
3. Describe the cultural context concerning GBV prevention and response. How do you think this may influence how GBV is prioritized and addressed in the humanitarian response?
   1. Prompt: cultural practices, social norms
4. What information is generated, managed and communicated? How?
   1. How is information and evidence used to inform the functioning GBV coordination system and prioritization and integration of GBV programs?
   2. Would you describe the flow of GBV coordination communication as bottom-up or top-down?

**Localisation**

1. What is the role of National NGO’s, Local Actors and civil society in the GBV coordination mechanism in Lebanon?
2. In your experience working with local actors are they real partnerships?

**Field-level Implementation - Tracer Services**

1. What services are available at field level?
2. Who is implementing which services? Names of organisations.
3. How does or does not GBV coordination translate into field-level implementation?
4. What, if any, results of GBV coordination in field level implementation are you aware of?
   1. If yes, are there relevant reports or other documents you could share with me?

**Resources and Future Direction**

1. Are GBV coordination resources sufficient in this protracted crisis?
   1. Briefly describe how resources are mobilized and distributed within the GBV coordination system
   2. How can mechanisms be more sustainable? Probes: innovation, multi-year funding, ...
2. Is sustainability being addressed in the protracted crisis?
   1. If yes, how is it being addressed?
   2. Prompt: Strengthening collaborations and partnerships, Building partnerships with local and national actors and organizations, academic institutions for research
3. How do you think sustainable GBV programs and coordination structures can be built in the protracted crisis?
   1. Probe: Resource allocation, Donor prioritization, Partnership with Local Actors
4. What if anything needs to change with the current system to ensure GBV response sustainability in the protracted crisis?
   1. What if anything already has changed?

**COVID-19 Pandemic**

1. How has the covid pandemic impacted your work?
2. How are coordination mechanisms being adapted in response to the pandemic?
   1. Has this negatively affected your work?

**Conclusion**

1. What important lessons could we learn and apply to other contexts?
2. Do you have anything else to add? Suggestions? Recommendations?
3. Are there other individuals or organizations you can suggest I speak with regarding the GBV coordination mechanism in Lebanon?
