# Supplementary material for: Gender based violence (GBV) coordination in a complex, multi-crisis context: a qualitative case study of Lebanon’s compounded crises (2019–2023)
Source: Confl Health. 2023 Oct 23;17:50. doi: 10.1186/s13031-023-00543-8 (PMC10591352; doi:10.1186/s13031-023-00543-8)
Supplement: Supplementary file 2 — Additional File 2: Table 2: Documents included in the analysis. [file 13031_2023_543_MOESM2_ESM.docx]

**Table 2: Documents included in the analysis**

| AUTHOR, YEAR | STUDY |
| --- | --- |
|  |  |
| Irish Consortium on GBV, 2019 (141) | Responding and Empowering GBV Services in Lebanon in Response to the Syrian Crisis |
| UNFPA, 2020 (26) | Evolution of gender-based violence and sexual and reproductive health services within the Syria crisis response 2017–2020 |
| ABAAD & UNFPA, 2020 (27) | Mapping gender-based violence programmes, services, and policies in Lebanon |
| Government of Lebanon and United Nations, 2021 (117) | Lebanon Crisis Response Plan 2017- 2021 (2021 update) |
| Government of Lebanon and United Nations, 2022 (113) | Lebanon Crisis Response Plan 2022-2023 |
| OCHA, Humanitarian Country Team and partners, 2021 (143) | Emergency response plan Lebanon 2021-2022 |
| UN Women, UNHCR, UNICEF, ABAAD, CARE, DRC, IRC, Intersos, 2020 (152) | Joint Assessment: Impact of COVID-19 on the SGBV Situation in Lebanon |
| Inter-Agency SGBV Task Force Lebanon, 2021 (153) | The Impact of Lebanon’s Fuel and Electricity Crisis on Sexual- and Gender-Based Violence Programming |
| UN Women, UNFPA, 2020 (138) | Violence against women in the time of COVID-19, Lebanon 2020 |
| UN Women, 2021 (128) | A rapid gender analysis of the August 2020 Beirut explosion: An intersectional examination |
| UNFPA, 2020 (154) | COVID-19 / UNFPA best practices and lessons learned in humanitarian operations in the Arab region. |
| Inter-Agency SGBV Task Force Lebanon, 2021 | GBV task force Meeting minutes April 2022 – November 2022 |
| GBViMS Lebanon, 2022 (139) | Gender-Based Violence Information Management System Analysis of an increase in GBV incidents against children Quarter 3—2021 |
